# Optimizing the Protein Fluorescence Reporting System for Somatic Embryogenesis Regeneration Screening and Visual Labeling of Functional Genes in Cotton

**DOI:** 10.3389/fpls.2021.825212

**Published:** 2022-01-07

**Authors:** Gai-Yuan Hu, Jia-Yi Ma, Fen Li, Jing-Ruo Zhao, Fu-Chun Xu, Wen-Wen Yang, Man Yuan, Wei Gao, Lu Long

**Affiliations:** ^1^State Key Laboratory of Cotton Biology, School of Life Sciences, Henan University, Kaifeng, China; ^2^State Key Laboratory of Crop Stress Adaptation and Improvement, Henan University, Kaifeng, China

**Keywords:** organelles, fluorescent protein, subcellular localization, promoter analysis, visual marker

## Abstract

Protein fluorescence reporting systems are of crucial importance to in-depth life science research, providing systematic labeling tools for visualization of microscopic biological activities *in vivo* and revolutionizing basic research. Cotton somatic cell regeneration efficiency is low, causing difficulty in cotton transformation. It is conducive to screening transgenic somatic embryo using the fluorescence reporting system. However, available fluorescence labeling systems in cotton are currently limited. To optimize the fluorescence reporting system of cotton with an expanded range of available fluorescent proteins, we selected 11 fluorescent proteins covering red, green, yellow, and cyan fluorescence colors and expressed them in cotton. Besides mRuby2 and G3GFP, the other nine fluorescent proteins (mCherry, tdTomato, sfGFP, Clover, EYFP, YPet, mVenus, mCerulean, and ECFP) were stably and intensely expressed in transgenic callus and embryo, and inherited in different cotton organs derive from the screened embryo. In addition, transgenic cotton expressing tdTomato appears pink under white light, not only for callus and embryo tissues but also various organs of mature plants, providing a visual marker in the cotton genetic transformation process, accelerating the evaluation of transgenic events. Further, we constructed transgenic cotton expressing mCherry-labeled organelle markers *in vivo* that cover seven specific subcellular compartments: plasma membrane, endoplasmic reticulum, tonoplast, mitochondrion, plastid, Golgi apparatus, and peroxisome. We also provide a simple and highly efficient strategy to quickly determine the subcellular localization of uncharacterized proteins in cotton cells using organelle markers. Lastly, we built the first cotton stomatal fluorescence reporting system using stomata-specific expression promoters (ProKST1, ProGbSLSP, and ProGC1) to drive Clover expression. The optimized fluorescence labeling system for transgenic somatic embryo screening and functional gene labeling in this study offers the potential to accelerating somatic cell regeneration efficiency and the *in vivo* monitoring of diverse cellular processes in cotton.

## Introduction

Fluorescent proteins (FPs) are bioluminescent proteins used as labeling markers in organisms owing to their energy transfer reactions that produce fluorescence without species restrictions or substrate requirements (Chalfie, 1995). As potential biomarkers, combined with high-resolution microscopy, FPs are widely utilized in the study of various biological processes. The establishment and application of fluorescence reporting systems has played a great role in research on *Arabidopsis* and other model plants, accelerating the elucidation of the dynamic nature of cellular networks in plant biology (Hilleary et al., 2018; Lu et al., 2019).

The first discovered green fluorescent protein (GFP), isolated from the jellyfish *Aequorea victoria* in 1962 (Shimomura et al., 1962), is a monomer protein composed of 238 amino acids with a molecular weight of 27 kD. GFP shows intense green fluorescence under an excitation wavelength of 488 nm (Shimomura, 1979). Since the 1990s, scientists have modified and optimized GFP fluorescence by inserting introns and changing its base composition or the amino acids of the GFP chromophore so as to improve the stability and fluorescence intensity of GFP (Heim et al., 1994; Heim et al., 1995). Later, Tsien’s research team developed blue fluorescent protein (BFP), cyan fluorescent protein (CFP), and yellow fluorescent protein (YFP) by introducing mutations that changed both the excitation and emission spectra of FPs (Shaner et al., 2005). Matz et al. (1999) isolated a red fluorescent protein (RFP) from the oral disk of coral (*Discosoma* spp.). RFP, a homolog of GFP has an emission wavelength of 583 nm (Matz et al., 1999). In subsequent studies, a series of optimized RFPs, including DsRed, mCherry, and tdTomato, were obtained by modifying RFP using mutagenesis or tandem fusion (Campbell et al., 2002; Shaner et al., 2004). So far, more than 800 FPs spanning an entire rainbow of fluorescence tags have been developed^1^.

In plant genetic transformation processes, the FPs that are expressed intensely and transformed stably are used to screen positive transgenic events owing to their fluorescence characteristics. Since GFP was first expressed in sweet orange protoplasts, GFP has been applied to a variety of monocotyledonous and dicotyledonous species (Niedz et al., 1995; Jin et al., 2012). Nishizawa et al. (2006) expressed DsRed2 in soybean and selected positive individuals by screening leaves and seeds showing fluorescence. Compared with GFP, plants expressing DsRed2 appeared red under white light, which was both easier and more convenient to observe. Yan’s research group developed a visual toolbox to enhance selection efficiency of positive transformants following maize genome editing. The toolbox contains CRISPR/Cas9 expression cassettes and a visible marker with expression driven by a tissue-specific promoter. In this system, seeds containing Cas9 are visibly red in natural light owing to DsRed2 without being affecting genome editing efficiency and plant development (Xu et al., 2021). This utilization of FP makes the selection of successful transformants cost-effective and rapid, which accelerates plant research and agricultural innovations.

The study of protein–protein interactions is the basis for understanding biological activities. Biotechnology, including bimolecular fluorescence complementation (BiFC) and fluorescence resonance energy transfer (FRET) (Kodama and Hu, 2012; Rao et al., 2014), has been developed to validate protein interactions based on the properties of fluorescence. FPs are small in molecular weight and can fuse with functional proteins without destroying the protein characteristics. Therefore, FPs are often used as visible markers that can specifically locate plant organelles (Nelson et al., 2007). For example, Touret et al. (2005) labeled endoplasmic reticulum (ER) with green fluorescence by fusing the ER signal peptide sequence KDEL with GFP. Using ultra-resolution microscopic imaging techniques to track fluorescence imaging, the contributions of the plasma membrane (PM) and ER in phagosome formation and maturation was monitored (Touret et al., 2005). Teper-Bamnolker et al. (2020) fused GFP with VPE, a key factor in programmed cell death (PCD), and expressed the fusion protein in potato. Carbon starvation and darkness treatments induced PCD symptoms in leaves, coupled with punctate fluorescence labeling translocation from the ER to the central vacuole through tonoplast engulfment. The use of FPs helped demonstrate for the first time that VPE translocates to vacuoles through the autophagy pathway (Teper-Bamnolker et al., 2020).

Fluorescent proteins have been widely used to visualize hormone signaling outputs or to track the temporal and spatial expression patterns of target genes by fusing FPs to promoters (DeBlasio et al., 2010; Ckurshumova et al., 2011). Visualization of auxin distribution during meristem and organ development *in vivo* has been enabled by the synthetic reporter DR5-driven FP expression (Ulmasov et al., 1997). Further, TCS:GFP was developed to visualize cytokinin output in root stem-cell specification during early embryogenesis; the TCS:GFP construct was able to specifically report phosphorelay output triggered by endogenous cytokinin receptors (Müller and Sheen, 2008). Such synthetic reporters have been applied to a variety of plants and now greatly contribute to plant research (Wu and Hong, 2021). KST1 is a protein with stomata-specific expression that is activated in guard cells following symmetric division of guard mother cells. The fluorescent label KST1 promoter has been shown to confer guard cell expression in both monocotyledonous and dicotyledonous species, providing a convenient tool to study the involvement of stomata in various stress conditions through visualization of the movement of fluorescence (Kelly et al., 2017).

Cotton (*Gossypium* spp.) is one of the most important cash crops worldwide and a major source of natural fiber and oil. However, there are relatively few reports of FPs applications in cotton. Half a century after its discovery, GFP was first utilized in cotton in 2012. By observing the fluorescence and brightness of GFP following transformation, researchers evaluate GFP as a visible screening marker confirming stable transformation of cotton plants (Jin et al., 2012). In 2018, genetically engineered DsRed2 was expressed in two cotton cultivars. The visible red of DsRed2 was observed in positive transgenic materials and stably transmitted to F_1_ and F_2_ progeny. The authors concluded that DsRed2 could be used as a visual marker for use in the evaluation of genetically modified lines (Sun et al., 2018). Yu et al. (2019) generated transgenic cotton with visualized cytoskeletons in their fiber by expressing GFP-labeled F-actin and RFP-labeled microtubules. The morphogenesis of cotton fiber and cytoplasmic trafficking at different developmental stages was then observed by live-cell imaging. This study demonstrated that fiber cells grow in a highly polarized manner that elongates via a unique tip-biased diffuse growth mode (Yu et al., 2019).

With the low somatic cell regeneration efficiency, it is time-consuming and laborious for cotton transformation. Visual fluorescence screening system is necessary in cotton. In addition, rapid advances in genome sequencing technology and bioinformatics have revealed numerous uncharacterized genes, increasing sequence availability to researchers and enabling them to develop efficient tools for gene function studies in cotton (Li et al., 2014; Zhang et al., 2015; Wang et al., 2019; Ma et al., 2021). In the last decade, a series of genes involving important traits have been cloned, including *MYB25* (Machado et al., 2009), *GRL*s (Liu et al., 2021), *GhSP* (McGarry et al., 2016), *GoPGF* (Ma et al., 2016), and *CGP1* (Gao et al., 2020), which control cotton fiber initiation, disease resistance, plant architecture and flowering, pigment gland formation, and gland pigmentation, respectively. The CRISPR system has allowed the utilization of the identified genes in cotton improvement and breeding (Gao et al., 2017; Long et al., 2018; Wang et al., 2018). However, the continuing challenge is ascribing biological mechanisms to these key genes. Developing visualization and biochemical tools using FP reporting systems is one of the most effective approaches. The stable expression of GFP and DsRed2 in cotton makes the wide application of fluorescent protein in cotton possible, but GFP is limited in cotton vegetative tissues because of the auto-fluorescence. Therefore, the DsRed2 was more competent compared to GFP. Nowadays, the reform and replace of fluorescent proteins are ongoing, which makes the available fluorescence labeling systems in cotton currently remain limited. To optimize protein fluorescence reporting systems in cotton by expanding the range of available FPs, we selected 11 FPs spanning four colors with distinct spectral characteristics and then expressed them in cotton. We identified 9 of the 11 FPs as stably and intensely expressed in cotton, as well as their inherence by T_1_ progeny. Further, we constructed transgenic cotton expression lines with *in vivo* organelle markers using selected FPs and then applied a simple and highly efficient strategy to quickly determine the subcellular localization of uncharacterized proteins in cotton cells. Lastly, we built a stomatal fluorescence reporting system using GFP labeling of stomatal cells in cotton. The optimized fluorescence labeling system of cotton we developed offers great potential for *in vivo* monitoring of diverse cellular processes in cotton and will thus accelerate research on the biology of cotton plants.

## Materials and Methods

### Vector Constriction

The coding sequence of selected *FP* genes (*mRuby2*, *mCherry*, *tdTomato*, *G3GFP*, *Clover*, *sfGFP*, *EYFP*, *YPet*, *mVenus*, *ECFP*, and *mCerulean*) were cloned and inserted into the binary vector pK7WG2.0 by BP and LR recombination reactions to generate overexpression vectors. All *FP*s were expressed under the control of the enhanced constitutive expression promoter 2 × CaMV35S (Long et al., 2020a). For fusion expression, the organelle markers were fused with mCherry at the N-terminus (mCherry:PTS1), C-terminus (PIP2:mCherry, MAN1:mCherry, γTIP:mCherry, COXIV:mCherry, and rubisco:mCherry) or centrally (WAK2:mCherry:HDEL) (Nelson et al., 2007). The expression cassettes of fusion protein were inserted into pK7WG2.0 for constitutive expression driven by a 2 × CaMV35S promoter.

For promoter analysis, the promoter region of genes with stomata-specific expression (GC1, KST1, and GbSLSP) was cloned as described in a previous publication (Plesch et al., 2001; Yang et al., 2008; Han et al., 2013; Kelly et al., 2017). The cloned promoters were inserted into the pK7WG2.0 vector to replace the original 2 × CaMV35S promoter and drive the expression of Clover. The primers used in vector constriction are listed in the Supplementary Table 1.

### Plant Transformation

For the stable transformation of cotton plants, the vectors were introduced into *Agrobacterium tumefaciens* strain LBA4404. The *Agrobacterium*-mediated cotton transformation was performed as described by Jin et al. (2006) using *Gossypium hirsutum* L. ‘YZ1’ explants. The explants were cultured and selected on culture medium containing 50 μg/mL kanamycin for the growth of transgenic positive calluses; then, the calluses were transferred to induction culture media for the induction of embryotic calluses and the production of positive transgenic plants. To transform tobacco (*Nicotiana benthamiana*) plants, the constructed vectors were introduced into *A. tumefaciens* strain GV3101, and the leaves of 3-week-old tobacco plants were infiltrated with *Agrobacterium* as described in our previous study (Long et al., 2020b).

For subcellular localization of target proteins, the cotyledons of T_1_ plants of the organelle marker lines were infiltrated with *Agrobacterium* carrying constructs with GFP-fused target proteins, or wild-type cotyledons were co-infiltrated with *Agrobacterium* carrying constructs of GFP-fused target proteins and RFP-fused target proteins. The cotyledons were incubated at 28°C in the dark for 12 h, followed by a 48–72 h culture period at 25/28°C (night/day). Then, the cotyledons were collected for protoplast isolation and fluorescence observation as described in our previous study (Long et al., 2020b).

### PCR Analysis

For T-DNA insertion analysis of T_1_ progeny, genomic DNA was isolated from 0.1 g fresh leaf samples with a DNA extraction kit (Tiangen Biotech, Beijing, China). For tdTomato expression analysis, 0.1 g fresh samples of cotton tissues were harvested for RNA extraction, and 1 μg of total RNA was used to synthesis cDNA (Long et al., 2019b). The reverse transcripts were diluted 50 times with ddH_2_O and stored at −20°C until subsequent PCR amplification. The *GhUB7* gene (*Gh_A12G1102*) was amplified as an internal control. Then, 500–700 bp fragments were amplified from FP genes with Taq DNA polymerase (Vazyme, Nanjing, China) to analyze the T-DNA insertion or FP expression. Thermal cycling for PCR was performed as follows: 95°C for 2 min, followed by 30 cycles of 95°C for 20 s, 58°C for 20 s, and 72°C for 30 s, and a final 3 min at 72°C. The primers used for PCR amplification were listed in Supplementary Table 1.

### Fluorescence Observation

The fluorescence in embryonic callus, stamen, pistil, leaf, and seed tissues of transgenic cotton or tobacco epidermal cells was observed with a zoom-stereo microscope (OLYMPUS, Tokyo, Japan). The fluorescence in protoplast, stomatal cell, fiber, leaf epidermal cell, and root samples of transgenic cotton was observed with a confocal electron microscopy (Nikon, Tokyo, Japan). Transgenic samples larger than 2 cm^2^ were observed using a Hand-held lamp (LUYOR, Shanghai, China), and images were captured using an EOS 800D camera (Canon, Beijing, China) with a fluorescent filter set (LUYOR) for green and red fluorescence observation.

Green or yellow fluorescence was observed with a filter set for excitation at 488 nm and emission at 500–550 nm; red fluorescence was observed with a filter set for excitation at 561 nm and emission at 570–620 nm; cyan fluorescence was observed with a filter set for excitation at 405 nm and emission at 425–475 nm.

### Protein Analysis

For protein extraction, 0.1 g leaf samples were quickly ground in liquid nitrogen, and 1 mL of plant protein extraction buffer was added to the ground samples, which were then iced for 15 min and centrifuged to isolate the supernatant (Gao et al., 2013). Then, 40 μL volumes of the supernatant were added to 10 μL of 5 × SDS-PAGE loading buffer, heated to 95°C for 5 min, and then stored at −80°C prior to further experiments. Then, 200 ng of extracted protein were electrophoresed using 10% SDS-PAGE gel and then transferred onto PVDF membranes. The PVDF membranes were washed three times with TBST buffer and incubated with anti-GFP rabbit antibody [1:10000 (*v*:*v*) dilution] at 60 rpm and 4°C overnight, followed with incubation in HRP-conjugated goat anti-rabbit IgG [1:10000 (*v*/*v*) dilution (Proteintech, Chicago, IL, United States)] for 1 h. The PVDF membrane was treated with ECL western blotting substrate and then detected with a chemiluminescence imaging system (EvolutionCapt, Fusion, Beijing, China).

## Results

### Overexpression of Candidate Fluorescent Proteins in Cotton

More than eight hundred FPs of various fluorescence colors have been developed so far. Because of the labor-intensive and lengthy procedures of cotton genetic transformation, it is highly impractical to test all FPs in cotton. In order to select suitable FPs for application in cotton, we considered a series of optical characteristics of the known FPs, such as excitation spectra (λ_ex_), emission spectra (λ_em_), and brightness. Based on the evaluated FP characteristics and whether the particular FP is widely used in plant research, we selected 11 FPs in four fluorescent colors in the present study (Supplementary Table 2). The FPs we selected are red fluorescent proteins (mRuby2, mCherry, and tdTomato), green fluorescent proteins (G3GFP, sfGFP, and Clover), yellow fluorescent proteins (EYFP, YPet, and mVenus), and cyan fluorescent proteins (mCerulean and ECFP).

The coding sequences of FPs were, respectively, cloned and inserted into the binary vector pK7WG2.0 to generate 2 × CaMV35S-driven expression cassettes. The constructed vectors were transiently expressed in tobacco leaves to verify the effectiveness for FP expression before being expressed in cotton. As shown in Supplementary Figure 1, all vectors produced bright and expected fluorescence in tobacco epidermal cells. The 11 FPs were further expressed in transgenic cotton created by *Agrobacterium*-mediated stable transformation, and we monitored the fluorescence intensity of each FP in transgenic materials during the whole tissue culture and plant regeneration process. According to our observations, the heterologous expression of all 11 FPs did not lead to adverse effects on plant morphogenesis or development.

### Stable Expression of Red Fluorescent Proteins and Green Fluorescent Protein in Transgenic Callus and Derived Tissues

**FIGURE 1:**
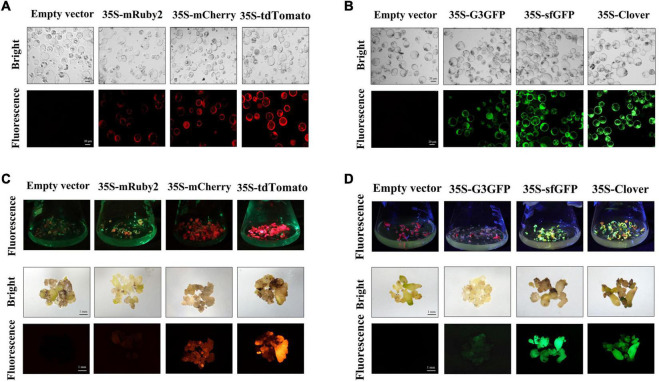
Stable expression of red and green fluorescent proteins (RFPs and GFPs, respectively) in cotton callus and embryo tissues. The protoplast of transgenic callus expression empty vector, RFP **(A)** and GFP **(B)** under white light (bright) and excitation light (fluorescence); scale bar, 20 μm. The transgenic embryo expression empty vector, RFP **(C)**, and GFP **(D)** under white light (bright) and excitation light (fluorescence); scale bar, 1 mm. RFPs: mRuby2, mCherry and tdTomato; GFPs: G3GFP, sfGFP and Clover.

The RFPs and GFPs were found to be stably expressed at the very early stage of the non-embryogenic callus formation. However, the brightness of these FPs in callus did differ. The fluorescence intensity of Clover, sfGFP, and G3GFP in calluses was consistently strong. However, for RFPs, the red fluorescence was stronger and more obvious for tdTomato in calluses, followed by mCherry, and mRuby2 fluorescence was relatively weak (Supplementary Figure 2). Protoplasts were isolated from the calluses and visualized under laser confocal microscopy. Most of the protoplasts expressed fluorescence (Figures 1A,B). These data indicate the high expression efficiency of the selected RFPs and GFPs in cotton callus, which could be available for transgenic callus screening.

The fluorescence in transgenic embryo was visualized at 4 months after transformation (Figures 1C,D). Both mCherry and tdTomato were still stably expressed during the embryogenesis stage, and the fluorescence intensity of tdTomato was significantly higher than that of mCherry at the same time. However, the fluorescence intensity of mRuby2 in embryo could hardly be detected. Both Clover and sfGFP can be stably expressed in transgenic embryo and have strong fluorescence intensity, while the G3GFP-expressing embryos exhibited pale green fluorescence, which was quite distinct from the bright green fluorescence of G3GFP in cotton calluses and transiently transformed tobacco. These results suggest that the fluorescence of mRuby2 and G3GFP in calluses was diminished during embryogenesis for an unknown reason, such that mRuby2 and G3GFP may not be suitable for applications in cotton.

**FIGURE 2:**
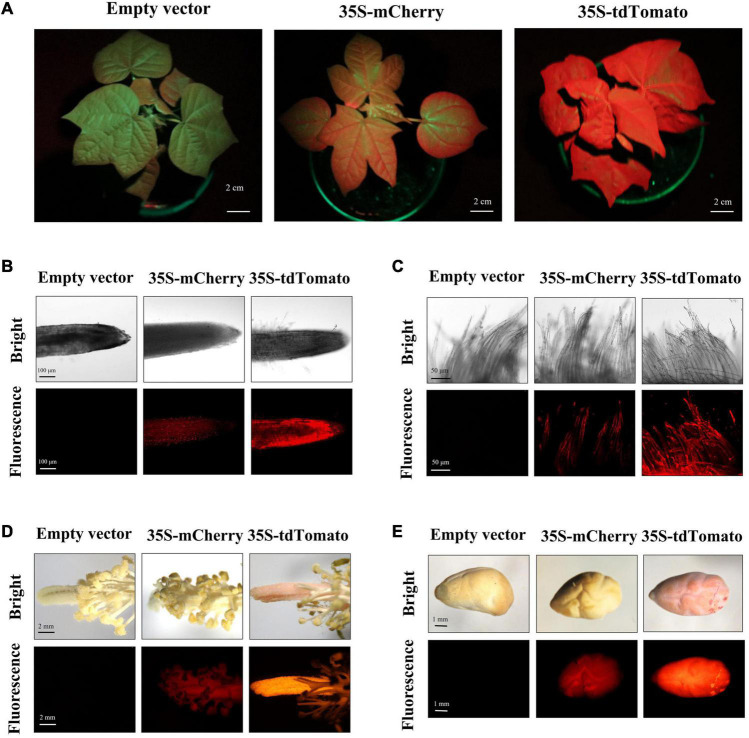
Red fluorescent protein (RFP) fluorescence in different tissues/organs of transgenic regenerated T_0_ plants. **(A)** Transgenic cotton plants expressing RFP during vegetative growth showed red fluorescence in their whole aerial portions under excitation spectra; scale bar, 2 cm. **(B–E)** The root (scale bar, 100 μm), fiber (scale bar, 50 μm), stamen/pistil (scale bar, 2 mm), and seed (scale bar, 1 mm) of transgenic regenerated cotton plants expressing empty vector and RFP under white light (bright) and excitation light (fluorescence).

**FIGURE 3:**
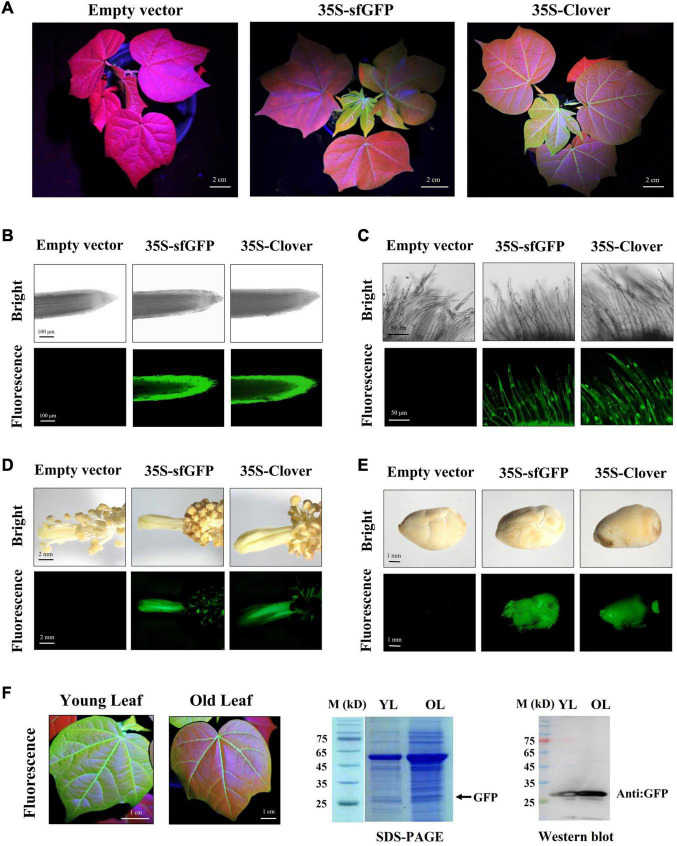
Green fluorescent protein (GFP) fluorescence in different tissues/organs of transgenic regenerated T_0_ plants. **(A)** Observation of green fluorescence under excitation spectra in aerial parts of transgenic cotton plants during vegetative growth stage; scale bar, 2 cm. **(B–E)** The root, fiber, stamen/pistil, and seed tissues of transgenic regenerated cotton plants under white light (bright) and excitation light (fluorescence). Scale bars (white) are defined in images. **(F)** Measurement of GFP in young and old leaf of transgenic cotton expressing Clover by western blot.

To detect the stability of FPs in regenerated cotton, the fluorescence of transgenic plants at different developmental stages was analyzed. During vegetative and reproductive stages, the whole plant of tdTomato transformants showed a bright red fluorescence, including leaf, petiole, root, stamen, pistil, fiber, and seed tissues (Figures 2A–E). Compared with the empty-vector-transformed cotton, the mCherry-expressing plants exhibited detectable red fluorescence expression in these tissues, but at a weaker level than that in tdTomato transformants. The green fluorescence of transgenic plants expressing Clover and sfGFP varied among tissues and organs. Although GFP expression was observed in vegetative organs, reproductive organs, and seeds (Figures 3A–E), the green fluorescence was predominant in young tissues and was diminished in older and chlorophyll-rich tissues (Figures 3A,F). The green fluorescence in the leaf veins of old leaves was similarly bright to that of young leaves, but the fluorescence intensity of mesophyll tissue in old leaves decreased significantly compared to young leaves. To investigate whether the decrease in fluorescence in old leaves was owing to the decrease in GFP level, we extracted the total proteins from young and old leaves and quantified their GFP contents. Western blots showed that more GFP protein was indeed accumulated in old leaves than in new leaves (Figure 3F). The above results indicate that the green fluorescence in older leaves was diminished, possibly owing to their higher chlorophyll content, which was consistent with previous studies in other species (Zhou et al., 2005; Hraška et al., 2006).

### Stable Expression of Yellow Fluorescent Proteins and Cyan Fluorescent Proteins in Transgenic Callus and Derived Tissues

**FIGURE 4:**
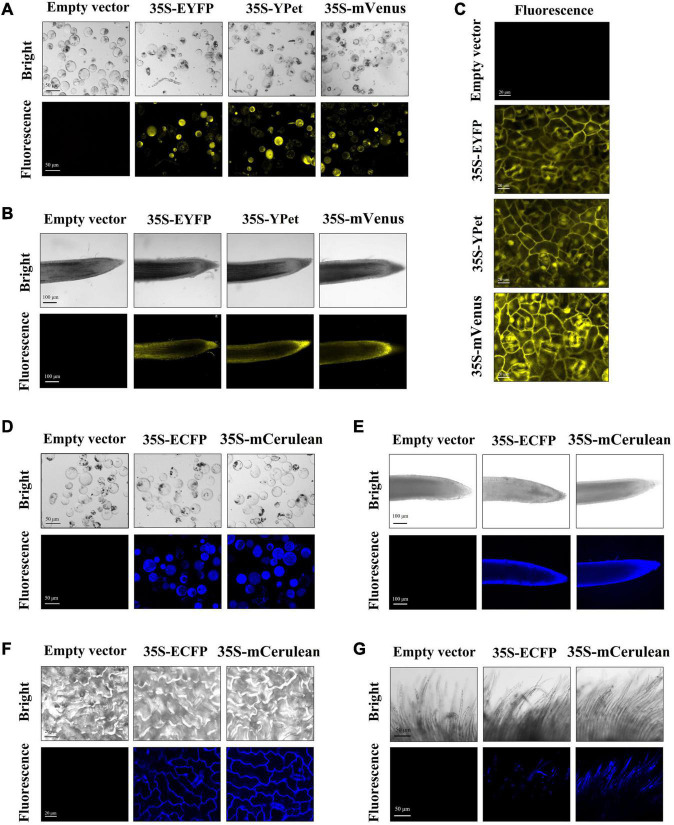
Yellow and cyan fluorescent protein (YFP and CFP, respectively) fluorescence in different tissues/organs of transgenic regenerated T_0_ plants. The yellow fluorescence observed in callus protoplast **(A)**, root **(B)**, and leaf **(C)** of empty-vector and YFP-expressing transgenic cotton. YFPs: EYFP, YPet, and mVenus. The cyan fluorescence observed in callus protoplast **(D)**, root **(E)**, leaf **(F)**, and fiber **(G)** tissues of empty-vector and CFP-expressing transgenic cotton. CFPs, ECFP and mCerulean. Scale bars (white) are defined in images.

Both YFPs (EYFP, YPet, and mVenus) and CFPs (mCerulean and ECFP) were also overexpressed in cotton constructed by *Agrobacterium*-mediated stable transformation. Owing to the limited availability of observation equipment, we could only observe the yellow and cyan fluorescence by confocal electron microscopy in this study. Therefore, the fluorescence in transgenic callus tissues was monitored from isolated protoplasts. Stronger yellow fluorescence was observed in callus protoplast samples expressing EYFP, YPet, or mVenus, and cyan fluorescence was observed in callus protoplast samples expressing mCerulean and ECFP (Figures 4A,D). We also monitored the fluorescence intensity of YFPs and CFPs in different organs of transgenic cotton. The yellow fluorescence was easily observed in roots and leaves, while mVenus yielded slightly stronger fluorescence than EYFP and YPet (Figures 4B,C). Similarly, stable cyan fluorescence was detected in roots, leaves, and fibers of transgenic cotton expressing ECFP and mCerulean (Figures 4E–G).

### Inherence of Fluorescent Proteins in T_1_ Plants

**FIGURE 5:**
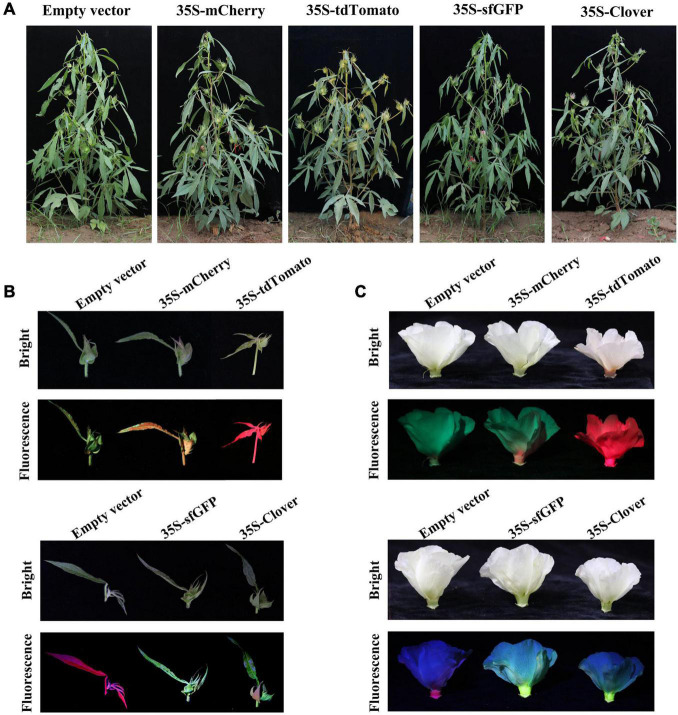
The fluorescence of T_1_ progeny in field conditions. **(A)** The morphology of empty-vector and green/red fluorescent protein (GFP/RFP, respectively)-expressing T_1_ plants in field at the reproductive stage. **(B)** Young fruiting branches of empty-vector- and GFP/RFP-expressing T_1_ plants growing in the field under white light (bright) and excitation light (fluorescence). **(C)** The flowers of empty-vector and GFP/RFP-expressing T_1_ plants under white light (bright) and excitation light (fluorescence). GFPs: sfGFP and Clover; RFPs: mCherry and tdTomato.

To analyze the stability and inheritance of the selected FPs in the offspring, transgenic cotton expressing FPs were self-pollinated, and the obtained seeds were planted in the field. The T_1_ progeny of FPs-expressing cotton exhibited similar morphological characters as the empty-vector-transformed cotton at various development stages (Figure 5A). The cotyledons of T_1_ individuals of transgenic lines for each FP were subjected to both fluorescence observation and subsequent PCR analysis to confirm T-DNA insertion. The transgenic positive T_1_ individuals exhibited fluorescence, while the negative individuals isolated from self-crossing T_1_ showed no fluorescence (Supplementary Table 3), indicating that fluorescence can indeed be inherited by subsequent generations and co-isolated with the inserted T-DNA. The fluorescence intensity in different organs of T_1_ plants was monitored, and both young shoots and flowers exhibited fluorescence in plants expressing mCherry, tdTomato, sfGFP, and Clover (Figures 5B,C). The yellow or cyan fluorescence was detected in young leaves of cotton expressing YFP (EYFP, YPet, and mVenus) or CFP (ECFP and mCerulean), respectively.

**FIGURE 6:**
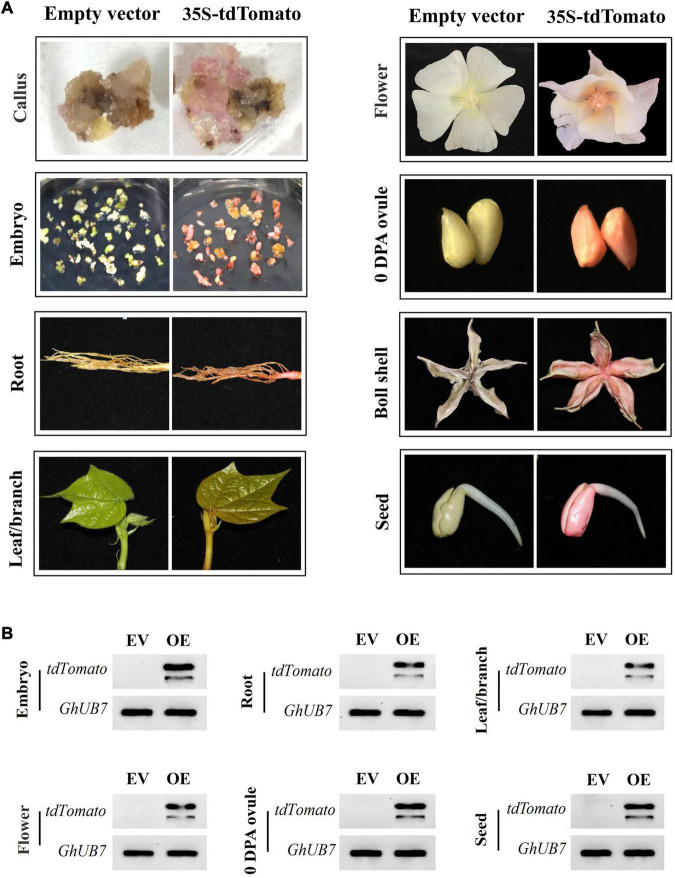
The tdTomato protein serves as a visual marker for successful transgenic events. **(A)** The constitutive expression of tdTomato led to pink coloration in different tissues/organs of transgenic cotton materials. **(B)** RT-PCR analysis of tdTomato expression of transgenic cotton materials appearing pink. EV, empty-vector-transformed cotton materials; OE, tdTomato-overexpressing cotton materials; *GhUB7*, the reference gene.

Notably, the transgenic materials expressing tdTomato were pink under white light compared to transgenic materials expressing empty vector, mRuby2, or mCherry. The pink color can be detected not only in callus and somatic embryos but also in various organs of mature plants, including root, leave, branch, flower, ovule, ball shall, and germinated seed tissues (Figure 6A). In addition, the heritability of the pink color was highly stable and associated with tdTomato expression (Figure 6B). These results provided further evidence of the stable and strong expression of tdTomato in transgenic cotton.

### Creation of Transgenic Cotton Expression Organelle Markers

It is widely recognized that the subcellular localization of protein is closely related to their functions. To construct transgenic cotton expressing *in vivo* organelle markers, mCherry was fused to a series of well-established organelle markers (Nelson et al., 2007). The constructed vectors were transient expressed in tobacco leaves to verify their effectiveness (Supplementary Figure 3) and then stably expressed in cotton through genetic transformation to produce organelle marker lines of cotton. The tested organelle markers covering seven specific subcellular compartments included the plasma membrane (PIP2), endoplasmic reticulum (WAK2/HDEL), tonoplast (γTIP), mitochondrion (COXIV), plastid (rubisco), Golgi apparatus (MAN1), and peroxisome (PTS1) (Supplementary Table 4).

**FIGURE 7:**
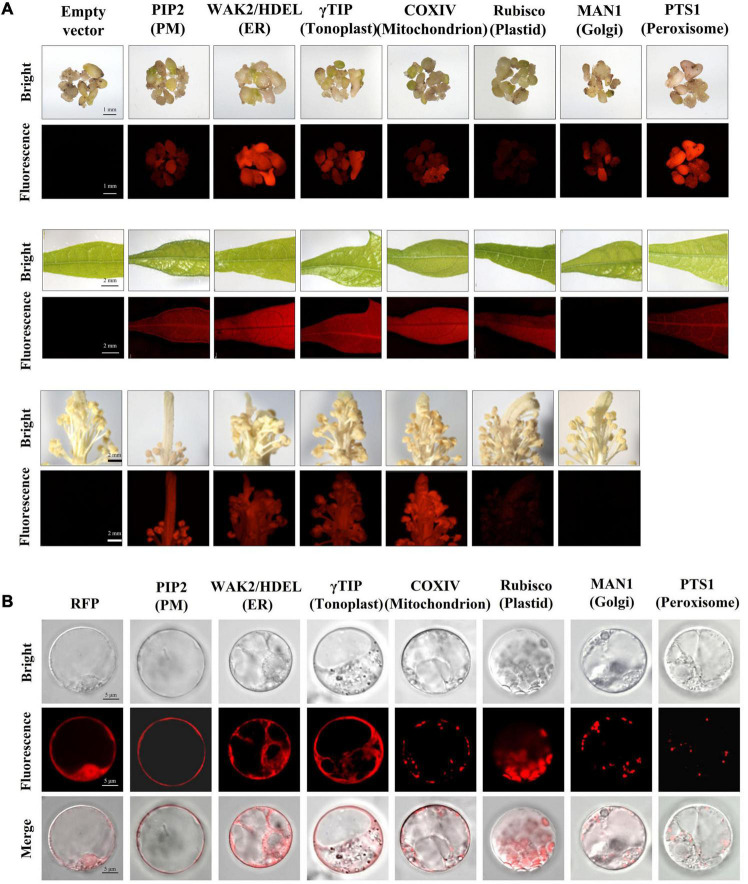
Transgenic cotton expressing *in vivo* organelle markers. **(A)** The transgenic embryo, leaf, and stamen/pistil expression of mCherry-fused organelle markers exhibited red fluorescence under white light (bright) and excitation light (fluorescence). Empty vector transformants were used as a negative control; scale bars: 1 mm in embryo observations, 2 mm in leaf and stamen/pistil observations. **(B)** Characteristic features of organelles in cotton protoplasts. The protoplast isolated from transgenic materials overexpressing mCherry as a positive control; scale bar, 5 μm. PM, plasma membrane; ER, endoplasmic reticulum.

The fluorescence of mCherry-fused organelle markers was monitored following transformation. Strong and stable red fluorescence could be detected in calluses, somatic embryos, and nutritional and reproductive organs of most transgenic organelle marker lines, with few exceptions. The fluorescence of rubisco:mCherry in callus, somatic embryo, pistil, and stamen tissues was relatively weak, which might be related to their low plastid production. In addition, wild-type-level fluorescence was detected in transgenic plants expressing MAN1:mCherry in leaf, pistil, and stamen tissues (Figure 7A). Unfortunately, most of the regenerated T_0_ plants that expressed mCherry:PTS1 had deformed flowers and failed to produce progeny. Otherwise, the transgenic plants with *in vivo* organelle markers showed no obvious differences from their wild-type counterparts in terms of growth and development, and the heritable progeny materials were obtained for further experiments.

To verify the subcellular localization of mCherry-fused organelle markers in cotton cells, the protoplasts of transgenic callus were isolated for fluorescence observation under laser confocal microscopy (Figure 7B). For rubisco:mCherry, the protoplasts were isolated from the cotyledons of T_1_ progeny because of the low levels of plastid contained in callus tissue. The mCherry alone was expressed in the nucleus and cytoplasm as a control. The red fluorescence of PIP2:mCherry targeting the PM was distributed as a single layer surrounding the cytoplasm on all sides. The WAK2:mCherry:HDEL construct was located along the ER, which has a sheet-like membrane and thin layer of cytoplasm continuous with the nuclear envelope. The tonoplast labeled by γTIP:mCherry included the membrane system around the vacuole and cytoplasmic tunnels. The COXIV:mCherry targets the mitochondrion, which is round and approximately 0.5 μm in diameter. The red fluorescence of rubisco:mCherry occurred in disk-like shapes, which coincided with the plastids distributed in the cells. MAN1:mCherry was distributed in Golgi bodies throughout the cytoplasm as small and round spots. The mCherry:PTS1 targeted peroxisomes, visualized as typical round structures varying in size from <0.5 to 2 μm in individual cells. These results indicate that the fluorescence distribution of these organelle markers in cotton cells can be identified by their characteristic morphologies and are consistent with those of model plants such as *Arabidopsis* and rice. The organelle markers have conserved subcellular localization patterns in dicotyledonous and monocotyledonous species, and the transgenic cotton expressing *in vivo* organelle markers we created are now available for subsequent research in cotton.

### Strategy for Fast Validation of Subcellular Localization of Target Proteins

**FIGURE 8:**
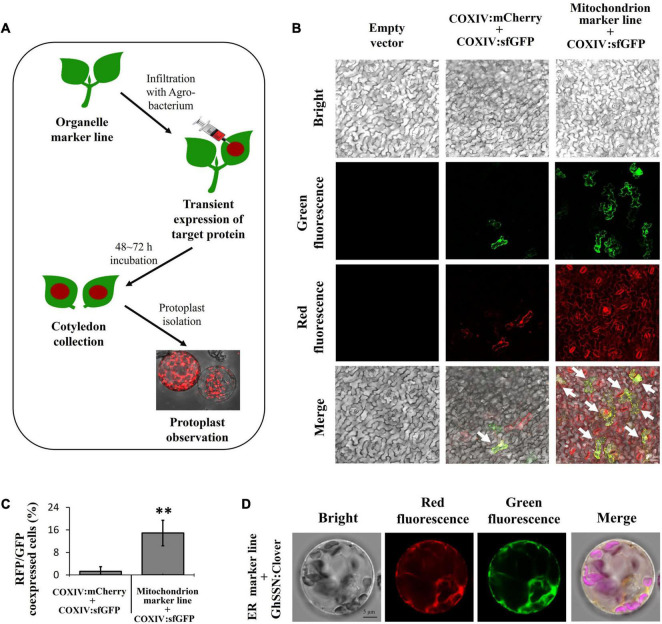
Fast validation for the subcellular localization of target protein in cotton cells. **(A)** Diagram of the strategy for validation of protein localization in cotton cells using transgenic cotton organelle marker lines. **(B)** Evaluation of the co-expression efficiency. Co-infiltration of COXIV:sfGFP and COXIV:mCherry constructs into cotyledons of wild-type cotton (middle panel, control group); infiltration of the COXIV:sfGFP construct into cotyledons of a mitochondrion marker line constitutively expressing COXIV:mCherry (right panel, experiment group). The cells expressing both sfGFP and mCherry are indicated with white arrows; scale bar, 20 μm. **(C)** The calculation of red and green fluorescent protein (RFP and GFP, respectively)-co-expressing cells in cotton cotyledons. *n* ≥ 5, **P < 0.01, *t*-test. **(D)** Subcellular localization of GhSSN. The protoplast was isolated from cotyledons of a cotton endoplasmic reticulum (ER) marker line infiltrated with GhSSN: Clover; scale bar, 5 μm.

Studies on subcellular localization of functional cotton proteins are usually conducted in *Arabidopsis* and tobacco owing to technical limitations (Gao et al., 2018; Long et al., 2020b). To facilitate localization experiments, we developed a convenient and simple system for subcellular localization of functional proteins in cotton cells. In this system, the target protein is fused with FPs other than RFP. The constructs containing the fusion protein are then introduced into *A. tumefaciens* and infiltrated into cotyledons of organelle marker lines for transient expression. After a 48–72 h incubation, cotyledons are harvested for protoplast isolation and fluorescence observation (Figure 8A).

To evaluate the efficiency of our system, the COXIV:sfGFP construct was infiltrated into cotyledons of a mitochondrion marker line that constitutively expresses COXIV:mCherry, and the COXIV:sfGFP construct co-infiltrated with the COXIV:mCherry construct in wild-type cotyledons was used as a control. The cotyledon cells expressing both red and green fluorescence were recorded. In the control group, most cells had no fluorescence, while a few had either green or red fluorescence. Only 0.35% of cells co-expressed COXIV:mCherry and COXIV:sfGFP. In the experiment group, red fluorescence was found in all cells from the transgenic organelle marker line. Green fluorescence was observed in 8.12% of the RFP-expressing protoplasts, and the fluorescence signals of COXIV:sfGFP and COXIV:mCherry were highly overlapping, demonstrating 23-fold efficiency of co-expression in our system compared to the control group (Figures 8B,C).

Furthermore, a cotton cytochrome P450 protein (SSN) that has previously reported been localized to ER of tobacco protoplasts was fused with the C-terminus of Clover (Sun et al., 2014). The SSN:Clover construct was infiltrated into cotyledons of transgenic organelle marker lines expressing WAK2:mCherry:HDEL. The protoplast was isolated from infiltrated cotyledons. The majority of green fluorescent signals overlapped with red fluorescence from the WAK2:mCherry:HDEL construct, indicating that SSN was localized to the protoplast ER (Figure 8D). The organelle marker lines combined with the fast and convenient strategy we have provided in this research enable quick and easy validation of the subcellular localization of uncharacterized proteins in cotton cells.

### Establishment of a Stomatal Fluorescence Reporting System

The FPs fluorescing in different colors are promising tools for studying gene expression patterns in plants, but they have rarely been implemented in cotton so far. In this study, specific genes were selected, and their promoters were cloned to drive the expression of the fluorescent proteins for use in tracking the spatial and temporal distribution. The partial promoter (642 bp) of *Solanum tuberosum KST1* was cloned owing to its constitutive expression in guard cells in at least eight plant species spanning monocots and dicots (Plesch et al., 2001; Kelly et al., 2017). Similarly, the promoter of a gene with guard-cell-specific expression, *GC1*, which encodes a small cysteine rich protein, was selected (Yang et al., 2008). In addition, the promoter of *GbSLSP* was cloned; *GbSLSP* encodes a subtilisin-like serine protease isolated from *Gossypium barbadens*e, which is preferentially expressed in guard cells and was verified using GUS and GFP as reporters in *Arabidopsis* and tobacco, but not in cotton (Han et al., 2013).

**FIGURE 9:**
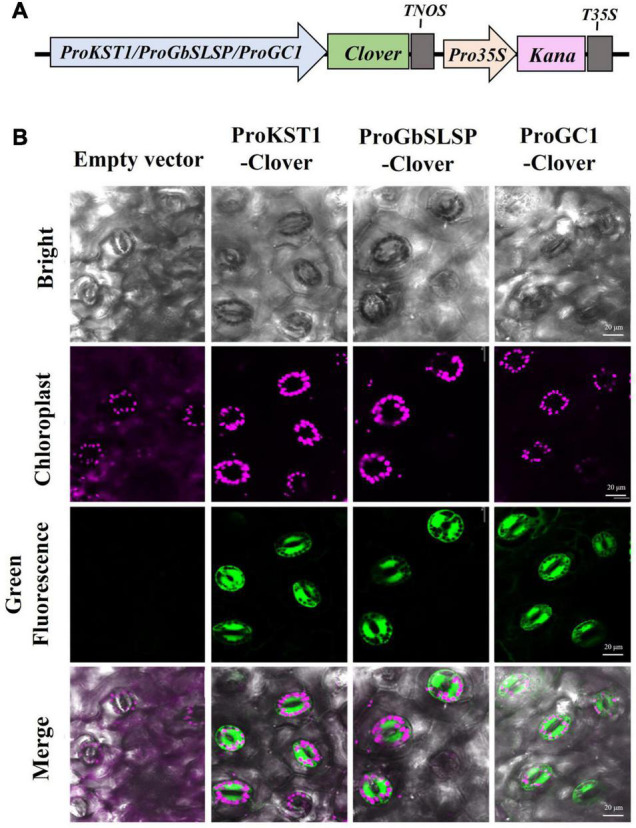
Green fluorescent protein (GFP) expression in cotton under the control of KST1, GbSLSP, or GC1 promoter, each of which is specific to guard cells. **(A)** Schematic diagram of specific promoter-driven expression constructs for genetic transformation of cotton. **(B)** Confocal images of leaf epidermal cells of transgenic cotton plants expressing Clover under the control of the KST1, GbSLSP, or GC1 promoter. The cotton expression empty vector was observed as a negative control, with chlorophyll autofluorescence shown in magenta; scale bar, 20 μm.

The cloned promoters (ProKST1, ProGbSLSP, and ProGC1) were, respectively, constructed into expression vectors, using Clover as a reporter gene and transform into cotton by *Agrobacterium*-mediated genetic transformation. To detect the stomata-associated fluorescence, the young leaves of the positive transgenic cotton were observed under laser confocal microscopy. In agreement with the results of research conducted in other species, it was found that green fluorescence was specifically distributed in the guard cells of transgenic cotton leaves expressing ProKST1-Clover, ProGbSLSP-Clover, or ProGC1-Clover, with no expression in mesophyll cells or roots (Figures 9A,B). Thus, we established a stomatal fluorescence reporting system in cotton.

## Discussion

The discovery and subsequent optimization of FPs are providing systematic labeling tools for visualizing microscopic biological activities *in vivo*, revolutionizing basic life science research. The use of spectrally distinct FPs has been of crucial importance to in-depth biological research, enabling multicolor imaging and the simultaneous visualization of multiple processes in anatomically inconspicuous cells or tissues (Luginbuehl et al., 2020). The established fluorescence reporting systems have usually been designed for animal models and then transferred to model plants, while their application in other plant species (such as cotton) has been limited. The main constraints of these systems are the uncertain functionality of FP among species, the high cost of system construction, and labor-intensive and lengthy transformation procedures. The aim of this research is to break through these technical barriers by selecting FPs that are stably and intensely expressed in cotton callus and derived transgenic tissues, and optimizing the fluorescence reporting system in cotton for further study. To achieve this goal, we screened 11 FPs covering four colors to be stably expressed in cotton. The FPs provide high fluorescence intensity and minimal interference with development during the whole cotton transformation and regeneration process. Further, we constructed the organelle marker lines and stomatal reporting system using the screened FPs.

Among the 11 selected FPs, mRuby2 and G3GFP were only expressed in dedifferentiated calluses, and their fluorescence signals were diminished after cellular differentiation. To our knowledge, G3GFP has been reported to be expressed in others crops (Dangol et al., 2017), contrasting with our results. The results indicate that the stability and fluorescence intensity of FPs may differ among species; hence, it is necessary to test the stability and brightness of FPs before their utilization in a particular species. Besides mRuby2 and G3GFP, the other nine FPs were stably expressed in regenerated cotton and heritable to T_1_ progeny, which provides us with nine FPs covering four different colors for applications as fluorescence reporting systems in cotton. The selected FPs offer great potential for the *in vivo* monitoring of diverse cellular processes in cotton, such as gene expression pattern, signal transduction, and protein transport and localization. Notably, the fluorescence intensity of a FP in different tissues of transgenic cotton could be unpredictable. Our experimental data showed that the strong fluorescence of YFPs (EYFP, YPet, and mVenus) and CFPs (mCerulean and ECFP) was observed in tissues such as root and leaf. However, owing to the limited availability of appropriate detection equipment, the possibility of unstable expression of these YFPs and CFPs in other tissues cannot yet be eliminated. Previous research has revealed that the green fluorescence of GFP is diminished in tissue with high chloroplast content owing to the auto-fluorescence of chlorophyl (Zhou et al., 2005; Hraška et al., 2006). This matches the observations of Clover and sfGFP expression in transgenic cotton and reminds researchers of the importance of choosing proper fluorescent labels for specific tissues. In particular, cotton is rich in secondary metabolites, so will the presence of a large number of metabolites affect the fluorescence observation? For example, cotton pigment glands store various amounts of known and unknown metabolites, causing the pigment gland tissue to be shown in black/brown color (Gao et al., 2020), and the fluorescent protein chosen to be expressed in pigment glands thus requires more comprehensive consideration. In contrast with GFP, the expression of mCherry and especially tdTomato was more stable among cotton tissues, and tdTomato had high fluorescence intensity in all tissues and organs of transformants and their offspring. In addition, tdTomato is easy to observe using different types of equipment, which makes tdTomato the optimal choice in a cotton fluorescence reporting system. However, using tdTomato as a visual marker in cotton still has some limitations. The protein size of tdTomato is twice that of other FPs (Supplementary Table 2), and thus, the possibility of affecting the proper folding of the target protein should be considered.

Additionally, tdTomato was readily detected not only under excitation light but also under natural light. The pink color of tdTomato was easily monitored throughout the very early stage of callus formation up to the last stages of cotton reproduction and was then transmitted to progeny. Owing to its stability across various tissues/organs and environments, tdTomato could be evaluated as a visual reporter for cotton genetic transformation. The explants of cotton transformation usually include hypocotyl, embryogenic callus, and shoot tip (Mccabe and Martinell, 1993; Jin et al., 2005; Jin et al., 2006). False positive and chimeric transformants are common phenomenon in genetic transformation of embryogenic callus and shoot tips; therefore, stable visible markers are crucial for positive transgenic screening in transformation procedures (Miki and Mchugh, 2004; He et al., 2020). The reporters used to visualize transformation usually include β-glucuronidase (GUS), anthocyanins, betalains, and FPs (Deng et al., 2012; Aliaga-Franco et al., 2019; Long et al., 2019a; He et al., 2020). Among the aforementioned reporters, FPs are cost-effective and stable across different cell types, and they have been widely used as visual markers to select desired traits. Here, we provided a convenient alternative to the existing visual markers; tdTomato allows for transformants and their offspring to be easily identified without any need for special equipment, thus increasing transformation efficiency by reducing screening workload and production cost.

Determination of subcellular localization can help to reveal the functional roles of proteins. Fluorescent organelle markers contain indicators for indistinguishable organelles that have been used to facilitate the precise determination of the subcellular localization of unknown proteins in plant species such as *Arabidopsis*, tobacco, maize, and rice (Nelson et al., 2007; Krishnakumar et al., 2015; Dangol et al., 2017). In this study, we have generated transgenic fluorescent organelle marker lines covering seven major organelles (PM, ER, tonoplasts, mitochondria, plastids, Golgi apparatus, and peroxisomes) (Nelson et al., 2007). The marker lines should be a valuable resource for localization studies conducted in cotton cells. In addition, organelle marker lines of cotton can be used to determine the distribution and dynamics of organelles under various environmental conditions. Transient expression is a rapid and consistent approach to studying protein subcellular localization and protein interactions. However, the efficiency of simultaneously expressing multiple proteins using transient expression has been relatively low in cotton. To address this, a convenient and simple system for subcellular localization of functional proteins in cotton was developed using organelle markers lines combined with transient expression of target proteins. This system elevated the protein co-localization efficiency 23-fold compared to the method of transiently expressing two FP-fused proteins simultaneously. The reliable and effective organelle marker lines and thus strategy for fast validation of the subcellular localization of target protein that we provide in this study will greatly facilitate the analysis of protein localization and organelle dynamics in cotton.

The GUS has been used as a reporter to study promoters in cotton in the past decade (Deng et al., 2012; He et al., 2018), but GUS detection requires toxic substrates and cofactors, which can be destructive to plant material and limit *in vivo* and real-time observation, thus affecting the reliability of experimental results. In contrast, FPs can be easily detected owing to their stable and strong fluorescence without any required substrates, enabling non-invasive monitoring and quantification of the spatiotemporal dynamics of gene expression to a single-cell-level *in vivo*. The use of FPs as cell- and tissue-specific markers has been widely applied in many plant species but not cotton (Ckurshumova et al., 2011; Hilleary et al., 2018; Lu et al., 2019). We provided nine FPs with four colors as candidate markers for the study of cotton promoters. Moreover, we have constructed a cotton stomata reporting system using three different stomatal specific expression promoters (ProKST1, ProGbSLSP, and ProGC1) identified in other species to drive the expression of one of the candidate FPs (Clover) (Plesch et al., 2001; Yang et al., 2008; Han et al., 2013). To the best of our knowledge, this is the first stomata fluorescence reporting system developed in cotton. This system can be used to study stomata development and their dynamics in cotton grown across different environments. These FPs help rapid screening transgenic callus and derived tissues, enable the non-invasive monitoring of gene expression, and offer great potential for new insights into gene function studies in cotton.

## Data Availability Statement

The original contributions presented in the study are included in the article/Supplementary Material, further inquiries can be directed to the corresponding author.

## Author Contributions

G-YH and J-YM analyzed and interpreted data and wrote the manuscript. FL performed the protoplast isolation. F-CX and J-RZ build the vectors. W-WY and MY helped to perform the cotton stable transformation. WG and LL designed the study and supervised all of work. All authors read and approved the final manuscript.

## Conflict of Interest

The authors declare that the research was conducted in the absence of any commercial or financial relationships that could be construed as a potential conflict of interest.

## Publisher’s Note

All claims expressed in this article are solely those of the authors and do not necessarily represent those of their affiliated organizations, or those of the publisher, the editors and the reviewers. Any product that may be evaluated in this article, or claim that may be made by its manufacturer, is not guaranteed or endorsed by the publisher.

## Footnotes

1
https://www.fpbase.org/table/
