# Supplementary material for: Optimizing the Protein Fluorescence Reporting System for Somatic Embryogenesis Regeneration Screening and Visual Labeling of Functional Genes in Cotton
Source: Front Plant Sci. 2022 Jan 7;12:825212. doi: 10.3389/fpls.2021.825212 (PMC8777222; doi:10.3389/fpls.2021.825212)
Supplement: Supplementary file 1 [file Data_Sheet_1.DOCX]

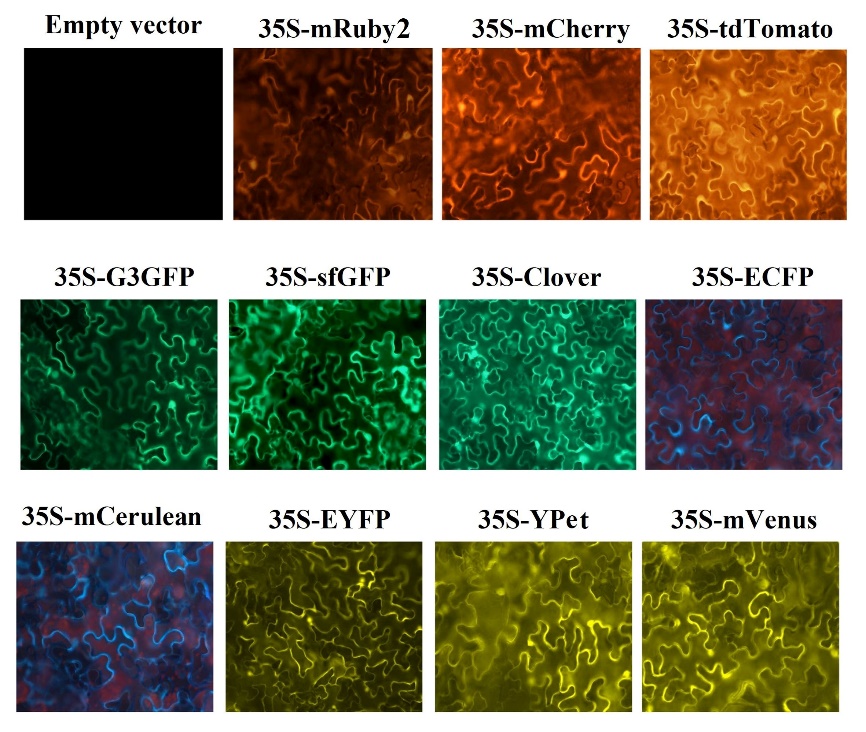


**Fig. S1.** Transient expression of red, green, cyan, and yellow fluorescent proteins (RFPs, GFPs, CFPs, and YFPs, respectively) in tobacco epidermal cells.


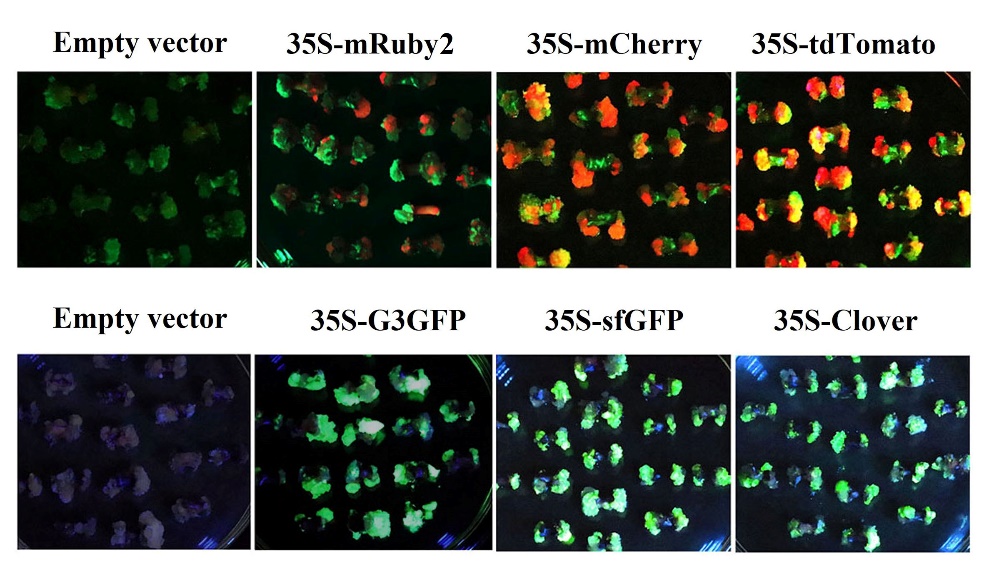


**Fig. S2.** Red and green fluorescent protein (RFP and GFP, respectively) fluorescence in stably transformed cotton calluses 2 months after transformation. Empty vector was transformed into cotton as a negative control. RFPs: mRuby2, mCherry, and tdTomato. GFPs: G3GFP, sfGFP, and Clover.


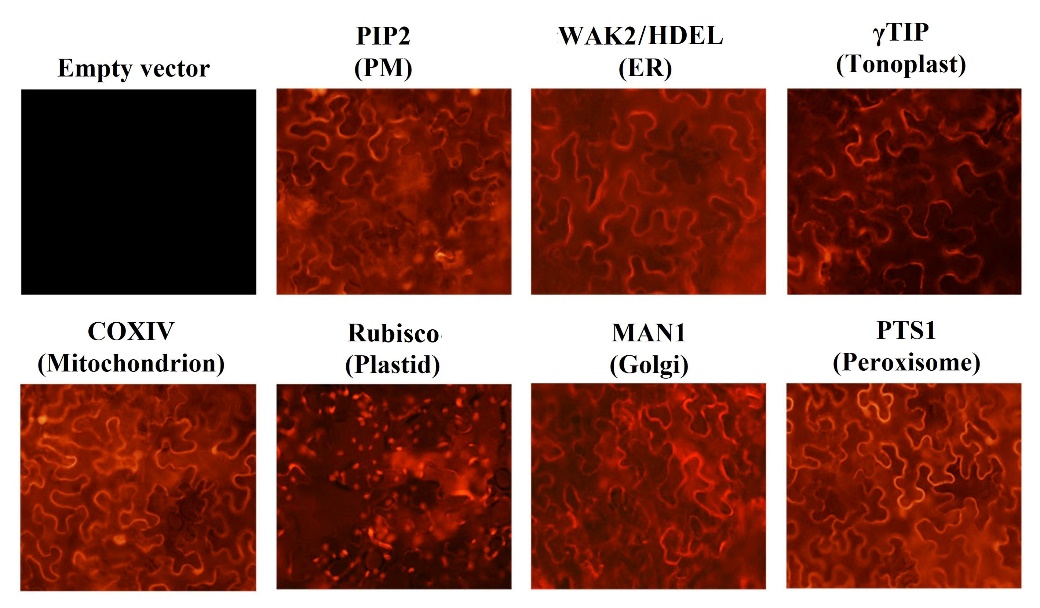


**Fig. S3.** Transient expression of mCherry-fused organelle markers in tobacco epidermal cells.
